# Propafenone is associated with fewer recurrences of supraventricular arrhythmias in mechanically ventilated patients with septic shock and right ventricular dysfunction

**DOI:** 10.3389/fcvm.2026.1807380

**Published:** 2026-05-07

**Authors:** M. Balik, T. Tencer, E. Svobodova, M. Otahal, I. Jurisinova, V. Kaspar, A. Krajcova, F. Duska, P. Waldauf

**Affiliations:** 1 Department of Anaesthesiology and Intensive Care, 1st Faculty of Medicine, Charles University and General University Hospital in Prague, Prague, Czechia; 2 Department of Anaesthesiology and Intensive Care, 2nd Faculty of Medicine, Charles University and University Hospital Motol, Prague Czechia; 3 Department of Anaesthesiology and Intensive Care, 3rd Faculty of Medicine, Charles University and Kralovske Vinohrady University Hospital, Prague, Czechia

**Keywords:** amiodarone, atrial fibrillation, echocardiography, propafenone, right ventricular dysfunction, septic shock, supraventricular arrhythmias

## Abstract

**Introduction:**

Supraventricular arrhythmias (SVA) are common in mechanically ventilated patients with septic shock and may be aggravated by right ventricular (RV) dysfunction. Optimal rhythm-control strategies in this setting remain uncertain. We explored whether propafenone differs from amiodarone in maintaining sinus rhythm in patients with RV dysfunction.

**Methods:**

This exploratory sub-analysis of the randomized double-blind PRASE trial included patients with septic shock who developed new-onset SVA, had preserved to moderately reduced left ventricular systolic function (ejection fraction >35%), and received continuous norepinephrine at doses <1.0 µg/kg/min. Patients were classified according to the presence or absence of RV dysfunction using comprehensive echocardiographic criteria. Outcomes included cardioversion at 24 h, arrhythmia recurrence, multiple recurrences, and 12-month survival.

**Results:**

Among 162 patients with sufficient echocardiographic data, 73 (45%) met criteria for RV dysfunction. Of these, 42 received propafenone and 31 amiodarone; 89 patients without RV dysfunction served as controls. Cardioversion at 24 h did not differ significantly between propafenone and amiodarone in patients with RV dysfunction (69% vs. 56.7%, *p* = 0.30). However, arrhythmia recurrence occurred less frequently with propafenone (50%) compared with amiodarone (83.9%; *p* = 0.003; OR 0.19, 95% CI 0.06–0.56). Multiple (>3) recurrences were also less common with propafenone (*p* = 0.028; OR 0.24, 95% CI 0.06–0.84). RV dysfunction was not associated with worse 12-month survival compared with controls, and no significant survival difference between antiarrhythmic strategies was observed.

**Conclusions:**

In mechanically ventilated patients with septic shock and RV dysfunction, propafenone was associated with fewer arrhythmia recurrences compared with amiodarone, despite similar short-term cardioversion rates. These findings are hypothesis-generating and suggest that propafenone may represent a promising alternative to amiodarone for rhythm control in critically ill patients with right ventricular dysfunction, warranting further prospective evaluation.

**Trial registration:**

ClinicalTrials.gov Identifier: NCT03029169.

## Introduction

Supraventricular arrhythmias (SVA) occur in the critically ill with an incidence between 8−25% and associate with two to five times increased mortality (1–3). Their incidence increases with the age, invasiveness of a surgical procedure, and the illness severity. For example, the rates of perioperative SVA hovers between 15% and 40% in cardiac surgery and can reach 46% in general ICU patients with septic shock (1–5).

Right ventricular (RV) dilatation, pressure overload, and pulmonary hypertension (PH) are strongly associated with the occurrence of an SVA (6). Across all forms of PH, the prevalence of supraventricular arrhythmias (SVA) ranges from 26% to 31% (7) with atrial fibrillation (AF) accounting for 67% (8). SVAs in PH cause hemodynamic deterioration that is disproportionate to the effects of the same arrhythmia in a normal heart. Loss of atrial kick and rhythm irregularity can significantly reduce RV stroke volume, decrease the pulmonary artery pressure (PAP), impair left ventricular filling, and ultimately reduce cardiac index. In this context, PH may no longer be readily detected due to uncoupling between the RV systolic function and pulmonary vascular resistance. The ratio of tricuspid annular plane systolic excursion (TAPSE), as a marker of RV systolic function, to systolic PAP typically decreases to below 0.3−0.4 and is usually accompanied by elevated RAP and venous congestion (9–11).

It is important to distinguish between pulmonary hypertension as a primary diagnosis and the broader syndrome of RV dysfunction encountered in mechanically ventilated patients with septic shock, where RV strain results from multiple concurrent mechanisms including elevated afterload from positive-pressure ventilation, volume overload, and direct stress/septic cardiomyopathy. There is an apparent scarcity of data from the intensive care setting. Nonetheless, in mechanically ventilated patients with septic shock, echocardiographically detected pulmonary hypertension (pulmonary artery systolic pressure ≥51 mmHg) after primary cardioversion has been associated with multiple recurrences of supraventricular arrhythmias (12, 13).

Across clinical scenarios, there is broad consensus that restoration and maintenance of sinus rhythm are particularly important in patients with pulmonary hypertension and supraventricular arrhythmias. New-onset atrial fibrillation (NOAF) in pulmonary hypertension is associated with a 2.1–4.7-fold increase in mortality when sinus rhythm cannot be achieved (14, 15). Current guidelines and expert opinion therefore advocate an early and proactive rhythm-control strategy in parallel with appropriate pulmonary hypertension-targeted therapies (6, 16, 17).

Initial management focuses on relieving RV strain through preload optimization, fluid unloading, and adjustment of mechanical ventilation to reduce right ventricular outflow impedance. In patients requiring aggressive ventilatory support, prone positioning may further alleviate right ventricular afterload (18). Bedside echocardiography is recommended to guide hemodynamic assessment and therapeutic decision-making (12, 19, 20).

Antiarrhythmic agents with minimal negative inotropic effects are generally preferred, and amiodarone is frequently used in this setting. However, in patients with severe right ventricular dysfunction and preserved left ventricular systolic function, the class Ic agent propafenone demonstrated a greater short-term benefit for rhythm control at 24 h compared with amiodarone, although this comparison was underpowered and did not reach statistical significance (19). Alternative strategies are limited by potential pulmonary toxicity of amiodarone and by the negative inotropic and chronotropic effects of beta-blockers in the presence of right ventricular failure. When rhythm control is unsuccessful or contraindicated, a rate-control strategy targeting a heart rate of 80–110 beats per minute is employed, typically using amiodarone, cautiously titrated beta-blockers when tolerated, or digoxin.

We hypothesized that in patients with septic shock and RV dysfunction defined by echocardiography, propafenone provides more effective rhythm control than amiodarone. To address this hypothesis, we performed an exploratory sub-analysis of the PRASE trial (19). focusing on arrhythmia-related outcomes in patients with right ventricular dysfunction. Patients without echocardiographic evidence of right ventricular dysfunction served as the comparator group.

## Methods

### Study design and population

This exploratory *post-hoc* analysis was conducted within the randomized controlled trial Propafenone Versus Amiodarone for Supraventricular Arrhythmias in Septic Shock (PRASE; ClinicalTrials.gov Identifier: NCT03029169), which enrolled mechanically ventilated critically ill patients with septic shock. The full study protocol and primary results of the PRASE trial have been published previously (21).

The PRASE trial recruited adult patients aged 16–85 years with septic shock defined according to Sepsis−3 criteria (22), preserved to moderately reduced left ventricular systolic function, and either new-onset supraventricular arrhythmia or new-onset supraventricular arrhythmia in patients with a history of paroxysmal supraventricular arrhythmias. According to the original study protocol, all eligible patients underwent baseline echocardiographic assessment and were subsequently randomized in a double-blind manner to receive either intravenous propafenone (bolus 35–70 mg followed by continuous infusion 400–840 mg/24 h) or intravenous amiodarone (bolus 150–300 mg followed by continuous infusion 600–1.800 mg/24 h). Crossovers to the alternative antiarrhythmic agent were negligible (3 patients of those 104 in the propafenone group and 6 patients of 105 in the amiodarone arm) (19), none of those were included in this exploratory analysis. Additional therapies (beta-blockers, magnesium, digoxin) were administered at the discretion of the treating physician and are summarized in eSupplementary Table S2.

Exclusion criteria included chronic persistent or permanent arrhythmias, prior MAZE procedure, pacemaker dependency, severe left ventricular (LV) systolic dysfunction (left ventricular ejection fraction (LVEF<35%), and ongoing norepinephrine infusion exceeding 1.0 µg/kg/min at the time of arrhythmia onset (21).

**Figure 1:**
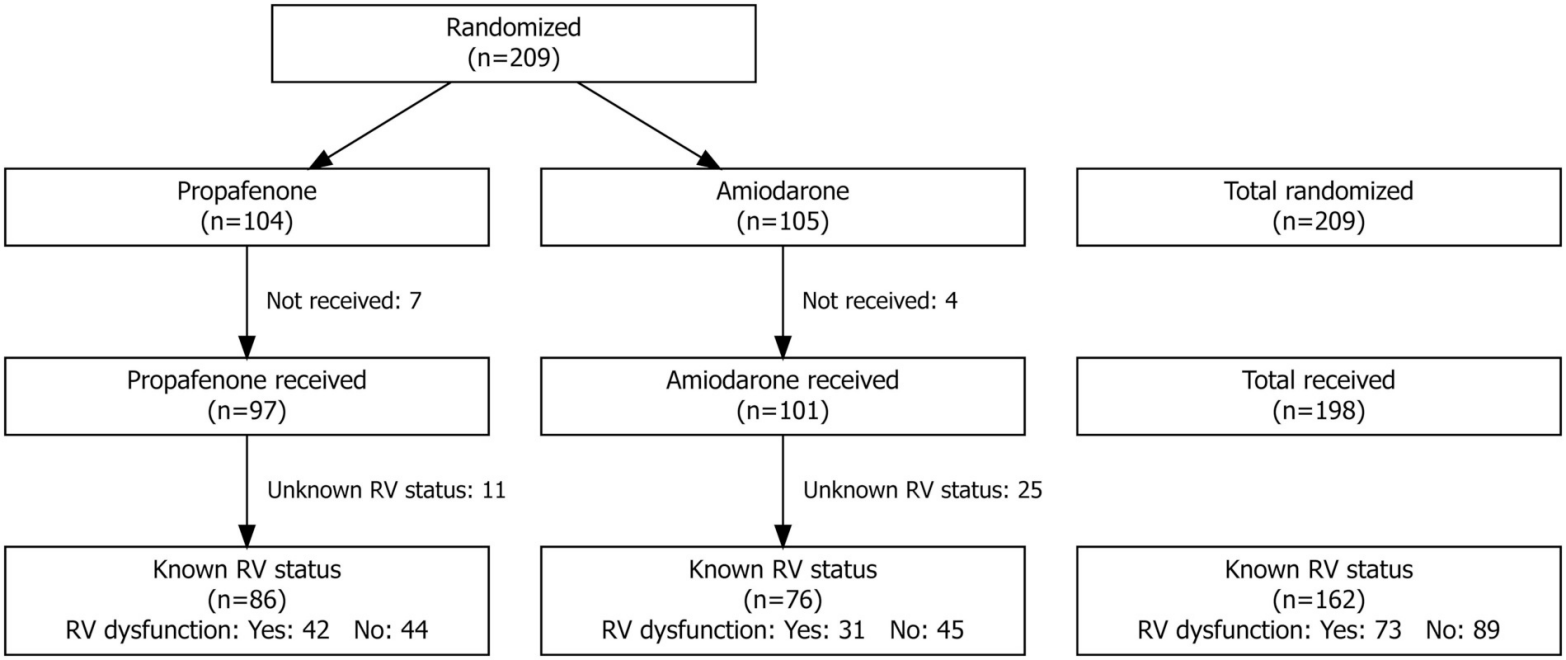
Study flow diagram. A total of 209 patients were randomized in the primary trial to receive propafenone (*n* = 104) or amiodarone (*n* = 105). Antiarrhythmic treatment was administered to 97 patients in the propafenone group and to 101 patients in the amiodarone group (7 and 4 patients, respectively, did not receive the assigned treatment). Right ventricular (RV) function could be assessed in 162 patients; echocardiographic assessment was not possible in 11 patients in the propafenone group and 25 in the amiodarone group, primarily due to inadequate acoustic windows. Among the 162 patients with assessable RV function, 73 patients were classified as having RV dysfunction (42 treated with propafenone and 31 with amiodarone), while 89 patients had no echocardiographic evidence of RV dysfunction (44 treated with propafenone and 45 with amiodarone).

According to the study protocol, patients underwent two additional echocardiographic examinations at 1 h and 4 h after cardioversion (Figure 1). The 4-hour post-cardioversion assessment was selected based on cardiology data suggesting recovery of left atrial mechanical function within 4–6 h after cardioversion (23–25).

The primary outcomes were arrhythmia recurrence and multiple recurrences occurring at any time after initial cardioversion during antiarrhythmic drug infusion. Successful cardioversion was defined as maintenance of sinus rhythm without recurrence of a sustained (>30 s) SVA. Arrhythmia recurrences were defined as any sustained SVA (>30 s) following initial cardioversion, adjudicated by the treating intensivist based on continuous 5-lead ECG monitoring, with 12-lead ECG confirmation every 12 h. Recurrences were categorized as one, two, three, or multiple (>3) events.

Data were collected using a study-specific electronic case report form (eCRF). Baseline patient characteristics included illness severity scores, source of septic shock, and hemodynamic and mechanical ventilation parameters recorded at arrhythmia onset.

At the time of arrhythmia onset, standard septic shock management was applied, including preload optimization, reduction of catecholamine infusions when feasible, electrolyte supplementation (targeting serum potassium >4.0 mmol/L and magnesium >1.0 mmol/L), and maintenance of adequate tissue oxygen delivery and lactate clearance. Hemodynamic monitoring consisted of standard intensive care unit monitoring (5-lead electrocardiography, invasive arterial blood pressure, central venous pressure, end-tidal carbon dioxide, and peripheral oxygen saturation), complemented by echocardiographic assessment.

Twelve-lead electrocardiography was performed every 12 h during antiarrhythmic drug infusion. Patients underwent pharmacological cardioversion while receiving the assigned antiarrhythmic therapy, with target heart rates of 80–110 beats per minute and mean arterial pressure above 70 mmHg. Electrical cardioversion was performed at any time in the presence of hemodynamic compromise, defined by signs of low cardiac output and/or inadequate pressure attributable to the arrhythmia.

Ethical approval for the study was obtained from the Ethics Committee of the First Faculty of Medicine and General University Hospital and from the Ethics Committee of the Third Faculty of Medicine and University Hospital Kralovske Vinohrady. Written informed consent was obtained from the patients’ next of kin. The conduct of the trial was regularly reported to the respective ethics committees, which served as the supervising research bodies, with mandatory progress reports submitted at least annually.

### Echocardiography

Echocardiography-guided optimization of preload was performed routinely and was mandatory in the presence of any hemodynamic instability. This approach was essential to avoid administration of the potentially cardiodepressant agent propafenone in the setting of sepsis-related deterioration of left ventricular systolic function.

All Doppler measurements were acquired at end-expiration and included three cardiac cycles during sinus rhythm at 1 h and 4 h after cardioversion, and 5–10 cardiac cycles at arrhythmia onset (time 0); measurements were subsequently analyzed and averaged. All recordings were obtained with simultaneous electrocardiographic monitoring (lead II) (26, 27).

The echocardiographic protocol included comprehensive left ventricular two-dimensional and Doppler assessment, focusing on ventricular filling, presence of active atrial systole confirming mechanical sinus rhythm, stroke volume, and cardiac index. Left ventricular ejection fraction (LVEF) was calculated using the biplanar Simpson method from apical four-chamber (A4C) and apical two-chamber (A2C) views, and the mean value was recorded. Left atrial volume and emptying fraction were measured at end-systole using the biplanar method in A4C and A2C views, with careful attention to measurement axis, and values were averaged (26, 28, 29).

Right heart assessment included right ventricular (RV) size and its proportion relative to left ventricular size, the presence or absence of paradoxical septal motion, tricuspid annular plane systolic excursion (TAPSE), and right ventricular fractional area change (FAC). The inferior vena cava (IVC) was evaluated for diameter and respiratory collapsibility. Pulmonary artery systolic pressure (PAPs) was estimated from the peak tricuspid regurgitant velocity.

RV dysfunction was defined by the presence of at least one of four combined two-dimensional and Doppler echocardiographic criteria (30, 31), as detailed in Table 1. In cases considered inconclusive (e.g., moderate RV dilatation), classification into the RV dysfunction or control group was guided by the presence or absence of venous congestion (32). Patients with mild RV dilatation were assigned to the control group (Table 1).

**Table 1:** Two-step echocardiographic definition of right ventricular dysfunction.

Echocardiography parameters (*n* = 110 patients)	RV dysfunction (*n* = 60)	Control group (*n* = 50)
PASP >40 mmHg and TAPSE <15 mm	✓	
PASP >40 mmHg and PSM	✓	
TAPSE <15 mm and PSM	✓	
RV/LV EDD ≥1.0	✓	

Additional echocardiography parameters in inconclusive patients (*n* = 52)	RV dysfunction (*n* = 13)	Control group (*n* = 39)
RV/LV EDD > 0.6 and < 1.0 and expiratory IVC diameter ≥ 20 mm with CI ≤ 15%	✓	
RV/LV EDD ≤ 0.6		✓

In the first step, right ventricular (RV) dysfunction was classified based on four predefined echocardiographic criteria in patients with complete data for all required parameters (*n* = 110). Patients meeting at least one of the four criteria were classified as having RV dysfunction (*n* = 60); those meeting none were assigned to the control group (*n* = 50). In the second step, patients with inconclusive classification due to moderate RV dilatation (RV/LV EDD ratio >0.6 and <1.0) were adjudicated by the presence or absence of venous congestion (*n* = 52): patients with an expiratory IVC diameter ≥20 mm and collapsibility index ≤15% were reclassified as having RV dysfunction (*n* = 13), while those without venous congestion were assigned to the control group (*n* = 39). Patients with only mild RV dilatation (RV/LV EDD ≤0.6) were assigned to the control group regardless of other parameters. Together, both steps allowed RV classification in 162 patients, corresponding to 82.7% of all patients enrolled in the trial. PASP, pulmonary artery systolic pressure; TAPSE, tricuspid annular plane systolic excursion; PSM, paradoxical interventricular septal motion; RV/LD EDD, right-to-left ventricular end-diastolic diameter ratio; IVC, inferior vena cava; CI, collapsibility index.

All echocardiographic examinations were performed by experienced operators trained in critical care echocardiography (Level II competency, EACVI or EACTA framework) according to a standardized acquisition protocol. The interobserver variability data are not available for this study. Echocardiographic images were uploaded and assessed by a single assessor (MB), who was blinded to the treatment assignment at the time of image review. RV dysfunction classification was therefore performed without knowledge of the randomization group.

### Statistical analysis

Statistical analyses were performed using R software (R Foundation for Statistical Computing, Vienna, Austria). Continuous variables were tested for distribution and are presented as median with interquartile range (IQR) or mean with standard deviation (SD), as appropriate. Categorical variables are expressed as counts and percentages. Comparisons between groups were performed using the Wilcoxon rank-sum test for continuous variables and Pearson's chi-squared test or Fisher's exact test for categorical variables, as appropriate.

Time-to-event analyses for achievement of cardioversion were conducted using Kaplan–Meier methods with censoring at 24 h, and groups were compared using the log-rank test. Twelve-month survival was analyzed using Kaplan–Meier curves with censoring at last follow-up, and comparisons were performed with the log-rank test.

Binary outcomes, including achievement of sinus rhythm at 24 h, occurrence of at least one arrhythmia recurrence, and occurrence of multiple (>3) recurrences, were further evaluated using unadjusted logistic regression models with treatment group (propafenone vs. amiodarone) as the sole predictor. Odds ratios (ORs) with 95% confidence intervals (CIs) were calculated. Absolute risk differences (ARDs) with 95% confidence intervals were calculated for all primary binary outcomes using the normal approximation method. Unadjusted models were used given the randomized design, which ensures treatment balance by allocation, and the limited subgroup sample size, which precludes stable multivariable modelling. Interaction analyses were performed to explore whether the effect of antiarrhythmic treatment differed according to right ventricular dysfunction status.

All analyses were exploratory in nature. This sub-analysis was not pre-specified in the original trial protocol and was not powered for treatment comparison within the RV dysfunction subgroup; results should therefore be interpreted as hypothesis-generating only. No correction for multiple comparisons was applied; the increased risk of type I error is acknowledged. Missing data were not imputed, and analyses were performed on available cases only. A two-sided *p*-value <0.05 was considered statistically significant.

## Results

A total of 209 patients with septic shock were enrolled in the primary trial. The most frequent source of sepsis was respiratory infection (*n* = 139, 66.5%), followed by abdominal (*n* = 39, 18.7%), urinary tract (*n* = 11, 5.3%), wound (*n* = 10, 4.8%), soft tissue (*n* = 6, 2.9%), and neuroinfections (*n* = 2, 1.0%).

At arrhythmia onset, most patients presented with atrial fibrillation (75.6%), followed by atrial flutter (19.6%) and supraventricular tachycardia (4.8%). Cardioversion to sinus rhythm was achieved in 201 patients (96%) within a median of 6.0 h (interquartile range 1.8–15.6) from initiation of antiarrhythmic therapy. The median duration of antiarrhythmic drug infusion was 109.5 h (46.8–186.2). Electrical cardioversion was required in 12.4% of patients prior to initiation of antiarrhythmic therapy and in 36.4% during ongoing antiarrhythmic infusion. Eight patients (3.8%) never achieved cardioversion and remained in a rate-controlled SVA (19).

Following successful cardioversion, 65 patients (31.1%) maintained sinus rhythm without any arrhythmia recurrence, whereas 134 patients (64.1%) experienced at least one recurrence of SVA. Among patients with recurrent arrhythmia, the median time to first recurrence was 21.7 h (17.3–27.5).

Echocardiographic assessment was performed in all patients at inclusion, in 173 patients at 1 h, and in 187 patients at 4 h after cardioversion.

At least one of the four predefined echocardiographic criteria for right ventricular dysfunction was fulfilled in 60 patients (52.6%), while the remaining 50 patients were initially assigned to the control group (Table 1). Among patients with moderate right ventricular dilatation, the presence of venous congestion led to reclassification of 13 additional patients into the RV dysfunction group, increasing its size to 73 patients (35.0% of all trial participants). Conversely, absence of venous congestion in this subgroup resulted in assignment of 39 patients to the control group, which increased to a total of 89 patients (43.0% of all participants). Patients with only mild right ventricular dilatation were assigned to the control group.

Overall, the presence or absence of right ventricular dysfunction could be determined in 162 patients, corresponding to 82.7% of all patients enrolled in the trial (Figure 1). Of the 73 patients with right ventricular dysfunction, 42 received propafenone and 31 amiodarone. These patients were compared with 89 controls without echocardiographic evidence of right ventricular strain, of whom 44 were treated with propafenone and 45 with amiodarone (Figure 1). Baseline characteristics of the study population are summarized in Table 2.

**Table 2:** Baseline characteristics of the study population stratified by right ventricular dysfunction. .

Parameter		All patients *n* = 162	RV dysfunction *n* = 73	Control group *n* = 89
Amiodarone		76 (46.9%)	31 (42.5%)	45 (50.6%)
Propafenone		86 (53.1%)	42 (57.5%)	44 (49.4%)
Age (years)		70 (63, 76)	70 (63, 74)	70 (63, 77)
Female		63 (38.9%)	26 (35.6%)	37 (41.6%)
BMI		29 (26, 33)	30 (26, 33)	29 (26, 32)
SOFA		10.0 (8.0, 12.0)	10.0 (8.0, 11.0)	10.0 (8.0, 12.0)
APACHE II		25 (20, 30)	24 (19, 29)	26 (20, 31)
History of an SVA		33 (20.4%)	14.0 (19.2%)	19.0 (21.3%)
Arrhythmia type	AF	124 (76.5%)	58 (79.5%)	66 (74.2%)
	Flutter	32 (19.8%)	13 (17.8%)	19 (21.3%)
	SVT	6 (3.7%)	2 (2.7%)	4 (4.5%)
**EFLV (%)**		**56** (**49, 69)**	**53** (**49, 62)**	**60** (**49, 74)**
LAVI (mL/m^2^)		31 (26, 39)	29 (26, 38)	32 (25, 40)
Norepinephrine (*n* = 162) (µg/kg.min)		0.30 (0.16, 0.45)	0.30 (0.15, 0.50)	0.30 (0.16, 0.42)
**Arginine vasopressin (*n*** **=** **48) (IU/h)**		**2.00** (**2.00, 3.00)**	**3.00** (**2.00, 4.00)**	**2.00** (**1.00, 2.00)**
Dobutamine (*n* = 10) (µg/kg.min)		3.00 (2.50, 3.00)	3.00 (2.50, 3.00)	3.00 (1.00, 5.00)
**CVP (mmHg)**		**8.0** (**6.0, 11.0)**	**10.0** (**6.0, 12.0)**	**8.0** (**5.0, 10.0)**
IPPV		155 (95.7%)	69 (94.5%)	86 (96.6%)
NIV		6 (3.7%)	4 (5.5%)	2 (2.2%)
PEEP (cmH_2_O)		8.0 (6.0, 10.0)	8.0 (6.0, 12.0)	8.0 (6.0, 10.0)
Pplat (cmH_2_O)		23 (18, 26)	24 (18, 26)	23 (16, 26)
Pmean (cmH_2_O)		14.0 (12.0, 18.0)	14.0 (12.0, 20.0)	14.0 (12.0, 16.5)
**MV (L/min)**		**9.3** (**8.0, 11.2)**	**10.0** (**8.2, 12.0)**	**8.7** (**7.7, 10.95)**
**FiO_2_ (%)**		**50 (40, 60), 54** **±** **18**	**50 (40, 60), 57** **±** **19**	**50 (40, 60), 51** **±** **17**
Potassium (mmol/L)		4.30 (4.10, 4.60)	4.30 (4.10, 4.50)	4.30 (4.00, 4.70)
Calcium (mmol/L)		1.91 (1.79, 2.03)	1.94 (1.81, 2.03)	1.89 (1.78, 2.04)
Ionized calcium (mmol/L)		1.05 (0.94, 1.11)	1.05 (0.96, 1.12)	1.03 (0.94, 1.09)
Magnesium (mmol/L)		1.30 (1.10, 1.57)	1.22 (1.01, 1.54)	1.37 (1.11, 1.58)
Hb (g/L)		101 (89, 117)	99 (89, 115)	101 (89, 118)
WBC (10^3^/µL)		18 (13, 25)	18 (14, 23)	17 (13, 26)
pH arterial		7.36 (7.30, 7.41)	7.35 (7.30, 7.41)	7.36 (7.31, 7.41)
pO_2_ arterial (kPa)		11 (10, 14)	11 (9, 13)	11 (10, 14)
**pCO_2_ arterial (kPa)**		**7** (**6, 8)**	**7** (**6, 8)**	**6** (**5, 8)**
BE arterial (mmol/L)		2 (−2, 5)	1 (−3, 6)	2 (−2, 5)
HCO_3_^−^ arterial (mmol/L)		27 (23, 31)	28 (23, 32)	27 (23, 30)
Lactate arterial (mmol/L)		2.55 (2.20, 3.80)	2.50 (2.20, 3.80)	2.60 (2.20, 3.80)
Temperature ( °C)		36.9 (36.4, 37.6)	36.9 (36.3, 37.6)	37.0 (36.5, 37.6)
CRP (mg/L)		187 (102, 316)	240 (117, 321)	180 (97, 292)
PCT (nmol/mL)		3 (1, 17)	2 (1, 15)	4 (1, 19)

Data are presented as median (interquartile range) or number (percentage), as appropriate. Between-group comparisons were performed using the Wilcoxon rank-sum test for continuous variables and the Pearson *χ*^2^ test or Fisher's exact test for categorical variables. Statistically significant differences are shown in bold (left ventricular ejection fraction, *p* = 0.01; arginine vasopressin dose, *p* = 0.008; arterial PaCO₂, *p* = 0.048; minute ventilation, *p* = 0.02; fraction of inspired oxygen with added mean ± SD, *p* = 0.04; central venous pressure, *p* = 0.009). Extended baseline characteristics are provided in eSupplementary Tables S1–S2 in the Electronic Supplementary Material. BMI, body mass index; SOFA, Sequential Organ Failure Assessment; APACHE II, Acute Physiology and Chronic Health Evaluation II; SVA, supraventricular arrhythmia; EF, ejection fraction; LAVI, left atrial volume index; IPPV, invasive positive-pressure ventilation; NIV, non-invasive ventilation; PEEP, positive end-expiratory pressure; Pmean, mean airway pressure; Pplat, inspiratory plateau pressure; MV, minute ventilation; FiO₂, fraction of inspired oxygen; Hb, hemoglobin; WBC, white blood cell count; CRP, C-reactive protein; PCT, procalcitonin.

Patients with right ventricular dysfunction and supraventricular arrhythmias did not differ from controls with respect to age, sex, illness severity scores, arrhythmia type, or norepinephrine dose (Table 2; eSupplementary Table S1 ESM). LVEF was modestly lower in patients with right ventricular dysfunction compared with controls [median 53% [49–62] vs. 60% [49–74], *p* = 0.01], whereas left atrial end-systolic volume did not differ between groups.

Patients with right ventricular dysfunction required higher doses of arginine vasopressin [3.00 [2.00–4.00] IU/h vs. 2.00 [1.00–2.00] IU/h, *p* = 0.008] and had higher central venous pressure [10.0 [6.0–12.0] mmHg vs. 8.0 [5.0–10.0] mmHg, *p* = 0.009]. Positive end-expiratory pressure, plateau pressure, and mean airway pressure were similar between groups; however, patients with right ventricular dysfunction exhibited higher arterial PaCO₂ [7 [6–8] kPa vs. 6 [5–8] kPa, *p* = 0.048], higher minute ventilation [10.0 [8.2–12.0] L/min vs. 8.7 [7.7–10.95] L/min, *p* = 0.018], and higher fraction of inspired oxygen (FiO₂ 57 ± 19% vs. 51 ± 17%, *p* = 0.041) (Table 2; eSupplementary Table S1 in the ESM).

Comparisons of prior medication history, concomitant potentially proarrhythmogenic drugs, and additional antiarrhythmic therapies administered during the ICU stay revealed no relevant differences between the groups (eSupplementary Table S2 ESM).

Comparison of intervention characteristics revealed no significant differences between patients with right ventricular dysfunction and controls (Table 3). Time from ICU admission to arrhythmia onset, time to initiation of antiarrhythmic infusion, duration of antiarrhythmic therapy, and length of ICU stay were comparable between groups.

**Table 3:** Supraventricular arrhythmia and intervention characteristics.

Parameter	All patients *n* = 162	RV dysfunction *n* = 73	Control group *n* = 89
Time from ICU admission to arrhythmia onset (hours)	52 (13, 116)	48 (6, 103)	52 (19, 121)
Electrical cardioversion prior to inclusion	19 (11.7%)	6 (8.2%)	13 (14.6%)
Arrhythmia onset to start of antiarrhythmic infusion (hours)	2 (1, 5)	2 (1, 4)	2 (1, 5)
Arrhythmia onset to cardioversion (hours)	10 (4, 25)	10 (4, 24)	11 (4, 30)
Start of antiarrhythmic infusion to cardioversion (hours)	7 (2, 17)	5 (1, 16)	7 (2, 17)
Duration of antiarrhythmic infusion (hours)	119 (60, 198)	102 (49, 175)	122 (65, 212)
Cumulative volume of antiarrhythmic infusion (mL)	793 (399, 1.210)	685 (384, 1.159)	811 (409, 1.362)
Sinus rhythm at 24 h	108 (68.4%)	46 (63.9%)	62 (72.1%)
Arrhythmia recurrence after cardioversion	108 (66.7%)	47 (64.4%)	61 (68.5%)
Electrical cardioversion after inclusion	62 (38.3%)	32 (43.8%)	30 (33.7%)
ICU length of stay (days)	13 (8, 22)	14 (9, 24)	13 (8, 19)

Data are presented as median [interquartile range] or number (percentage), as appropriate. Between-group comparisons were performed using the Wilcoxon rank-sum test for continuous variables and the *χ*^2^ test or Fisher's exact test for categorical variables. Time-to-event analyses were evaluated using Kaplan–Meier estimates and log-rank tests, where applicable. The cumulative volume of antiarrhythmic infusion represents the total volume infused per patient in this blinded study.

.

Rates of electrical cardioversion prior to study inclusion (8.2% in patients with right ventricular dysfunction vs. 14.6% in controls) as well as additional electrical cardioversions performed during antiarrhythmic pharmacotherapy (43.8% vs. 33.7%, respectively) did not differ significantly. Cardioversion rates at 24 h were similar between groups (63.9% vs. 72.1%), as were rates of arrhythmia recurrence, which occurred in 64.4% of patients with right ventricular dysfunction and 68.5% of controls (Table 3).

**Figure 2:**
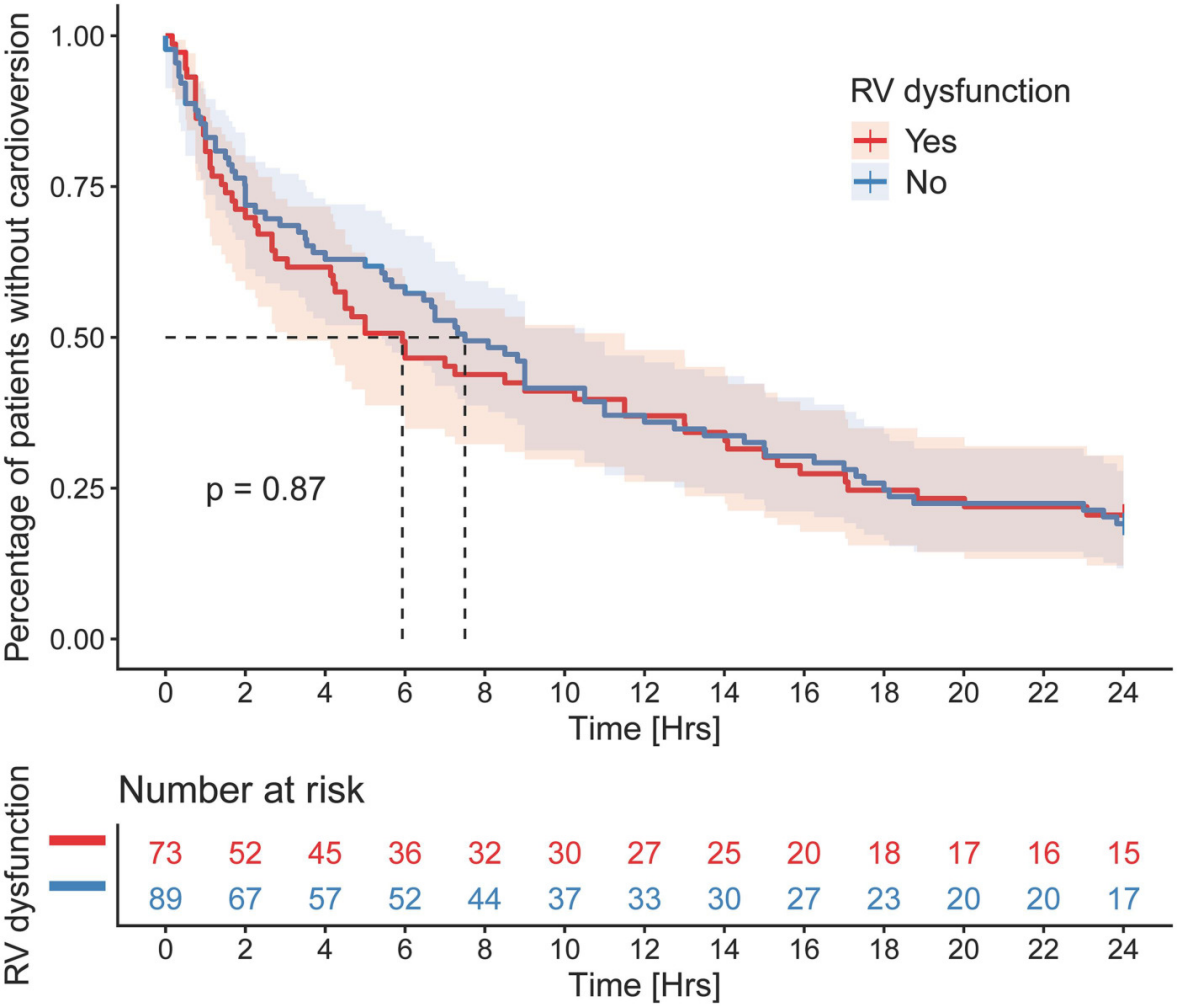
Kaplan–meier analysis of time to cardioversion. Kaplan–Meier curves comparing the proportion of patients who had not yet achieved cardioversion over the first 24 h after initiation of antiarrhythmic therapy in patients with right ventricular dysfunction and in controls. No significant difference between groups was observed over the 24-hour observation period (log-rank *p* = 0.87).

Patients with right ventricular dysfunction showed a trend toward faster cardioversion in the early period (3–8 h); however, no significant difference was observed over the 24-hour observation period (*p* = 0.87; Figure 2).

Right ventricular dysfunction was not associated with an increased risk of arrhythmia recurrence. At least one recurrence occurred in 64.4% of patients with right ventricular dysfunction compared with 68.5% of controls (*p* = 0.60). Similarly, no differences were observed in the distribution of one, two, three, or multiple (>3) arrhythmia recurrences between groups (36.2% vs. 31.1%, respectively; *p* = 0.80).

**Figure 3:**
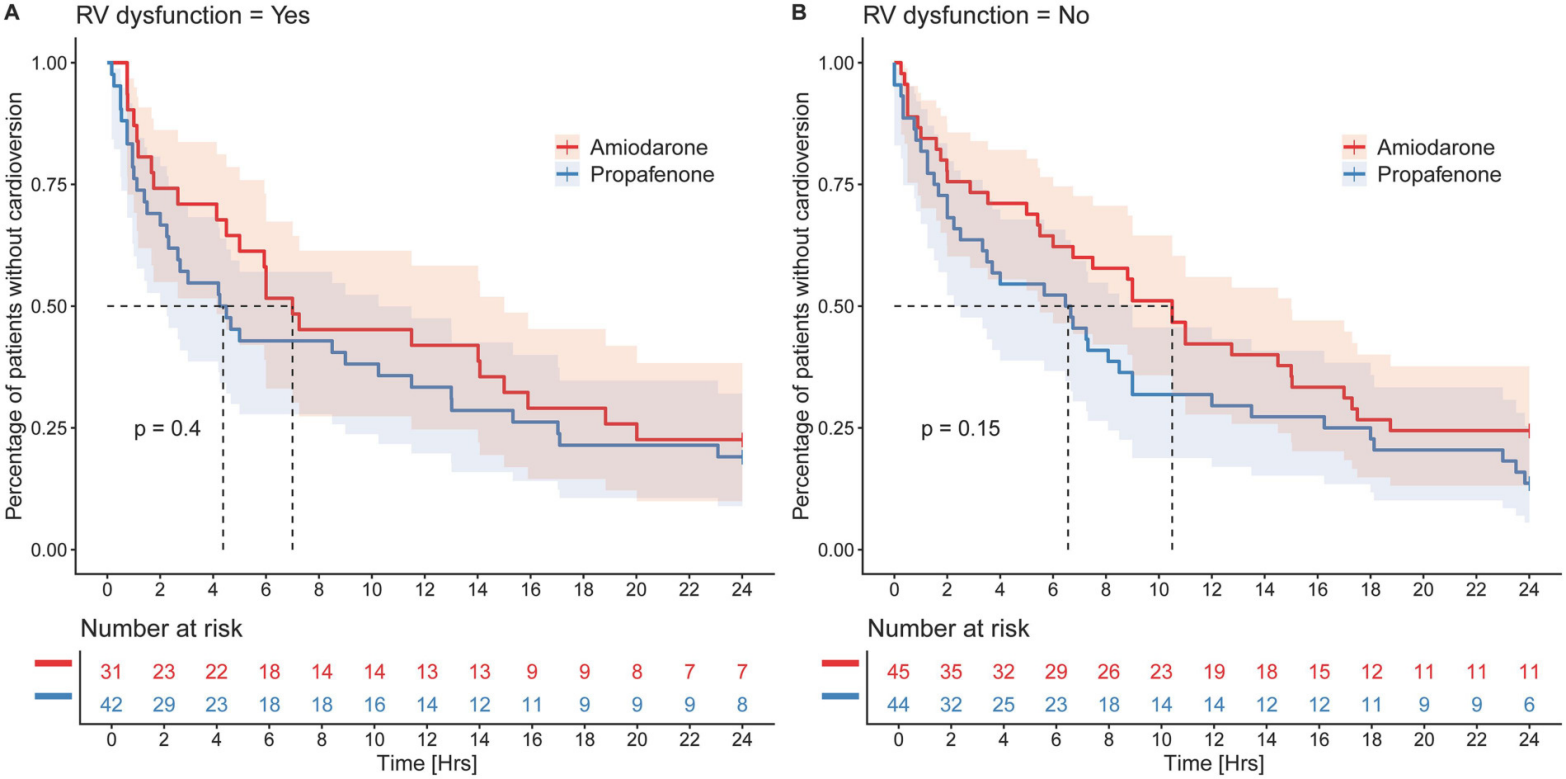
Kaplan–meier analysis of time to cardioversion stratified by treatment and right ventricular function. Kaplan–Meier curves comparing time to cardioversion between patients treated with propafenone and amiodarone in those with right ventricular dysfunction **(A)** and in controls without right ventricular dysfunction **(B)** No statistically significant difference between treatment groups was observed in either subgroup (log-rank *p* = 0.40 for patients with right ventricular dysfunction and *p* = 0.15 for controls).

Among patients with right ventricular dysfunction, achievement of sinus rhythm at 24 h was more frequent in those treated with propafenone than with amiodarone (69.0% vs. 56.7%); however, this difference did not reach statistical significance (*p* = 0.30). A similar lack of statistical significance was observed in the control group (75.0% vs. 69.0%, *p* = 0.50; Figure 3).

In the study group with right ventricular dysfunction, arrhythmia recurrence occurred in 50.0% of those treated with propafenone (*n* = 21) compared with 83.9% of patients treated with amiodarone (*n* = 26; *p* = 0.003). In contrast, no significant difference in arrhythmia recurrence was observed among patients without right ventricular dysfunction [61.4% [*n* = 27] with propafenone vs. 75.6% [*n* = 34] with amiodarone; *p* = 0.15].

Logistic regression analysis demonstrated that treatment with propafenone in patients with right ventricular dysfunction was associated with a significantly lower odds of arrhythmia recurrence compared with amiodarone (odds ratio 0.19, 95% confidence interval 0.06–0.56; *p* = 0.004). The absolute risk difference was −33.9 percentage points (95% CI −53.8% to −14.0%). No significant association between treatment strategy and arrhythmia recurrence was observed in patients without right ventricular dysfunction (odds ratio 0.51, 95% confidence interval 0.20–1.27; *p* = 0.15). An interaction analysis between RV dysfunction status and treatment assignment demonstrated that the effect of propafenone on arrhythmia recurrence did not reach statistical significance for a differential treatment effect across RV dysfunction strata in the primary analysis (interaction term OR 2.67, 95% CI 0.64−12.1; *p* = 0.185). In the sensitivity analysis using the strict RV dysfunction definition, the interaction was statistically significant (OR 9.26, 95% CI 1.61−63.3; *p* = 0.016), consistent with a greater treatment effect of propafenone relative to amiodarone in patients with RV dysfunction compared with those without (eSupplementary Table S3).

**Figure 4:**
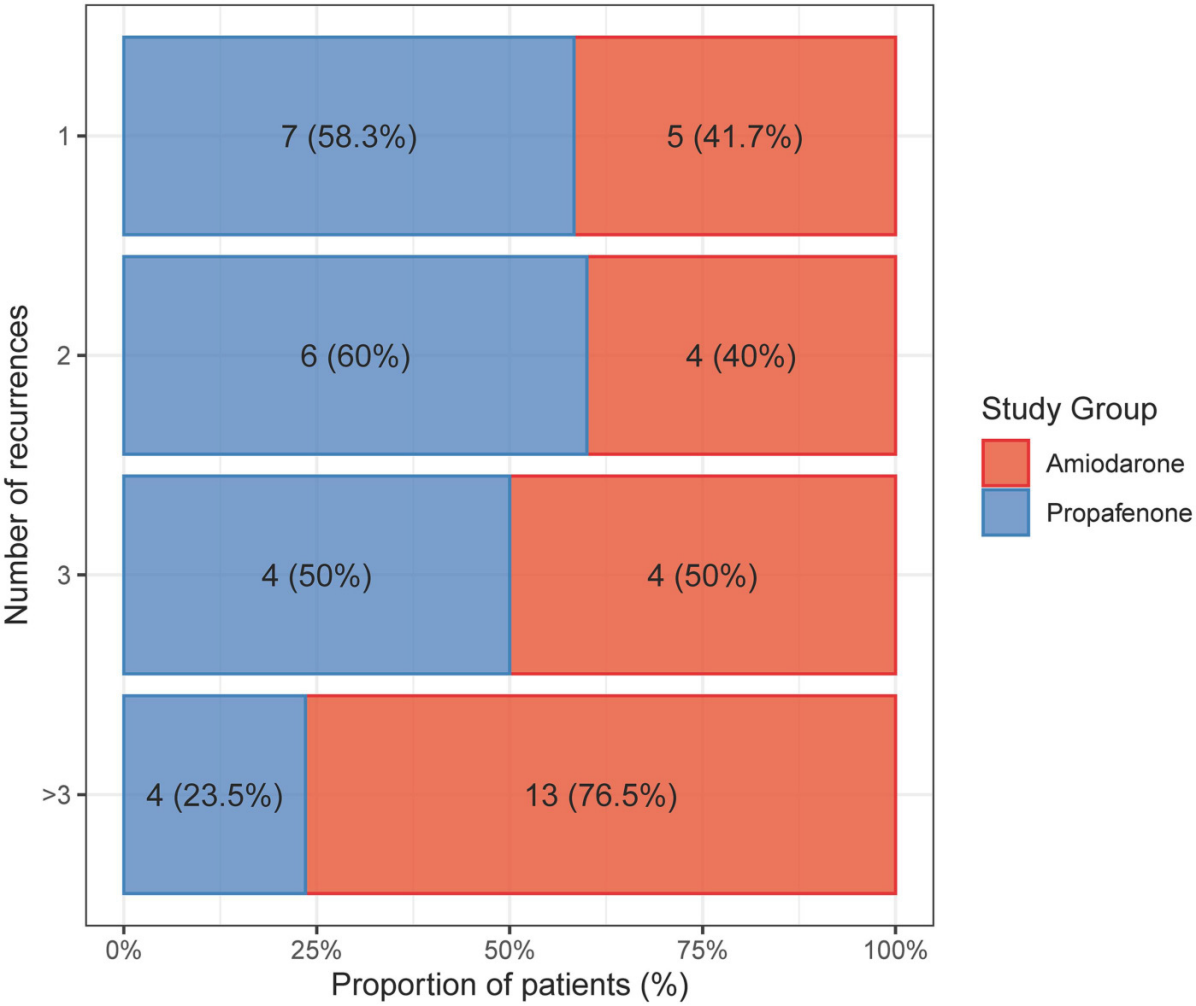
Distribution of arrhythmia recurrences in patients with right ventricular dysfunction according to antiarrhythmic treatment. The number and distribution of supraventricular arrhythmia recurrences differed between treatment groups, with fewer multiple (>3) recurrences observed in patients treated with propafenone compared with amiodarone. Logistic regression analysis demonstrated a lower odd of multiple recurrences with propafenone (odds ratio 0.24, 95% confidence interval 0.06–0.84; *p* = 0.03).

Among patients with right ventricular dysfunction who experienced arrhythmia recurrence (*n* = 47), treatment with propafenone was associated with fewer multiple (>3) arrhythmia recurrences compared with amiodarone. Multiple recurrences occurred in 19.0% of patients treated with propafenone (*n* = 4) and in 50.0% of those treated with amiodarone (*n* = 13; *p* = 0.028; Figure 4).

Logistic regression analysis confirmed that propafenone treatment in patients with right ventricular dysfunction was associated with a lower odd of multiple arrhythmia recurrences compared with amiodarone (odds ratio 0.24, 95% confidence interval 0.06–0.84; *p* = 0.03). The absolute risk difference was −31.0 percentage points (95% CI −56.5% to −5.4%). In contrast, no statistically significant association between treatment strategy and multiple recurrences was observed in patients without right ventricular dysfunction (odds ratio 0.32, 95% confidence interval 0.09–1.02; *p* = 0.06).

Survival analysis showed no significant difference in 12-month survival between patients with and without right ventricular dysfunction (*p* = 0.58). Similarly, no significant difference in 12-month survival was observed between patients treated with propafenone and those treated with amiodarone, either in the subgroup with right ventricular dysfunction (*p* = 0.09) or in controls without right ventricular dysfunction (*p* = 0.71).

As a pre-specified sensitivity analysis, the primary outcomes were re-evaluated using a strict RV dysfunction definition based on the four primary echocardiographic criteria only, without IVC-based adjudication for inconclusive cases. Under this definition, 60 patients met criteria for RV dysfunction (36 propafenone, 24 amiodarone) and 50 served as controls (26 propafenone, 24 amiodarone), with the RV status assessable in 110 patients (56.1% of those who received treatment). The direction and magnitude of the treatment effect on arrhythmia recurrence were consistent with the primary analysis and numerically stronger: recurrence occurred in 41.7% of propafenone-treated vs. 87.5% of amiodarone-treated patients with RV dysfunction (OR 0.10, 95% CI 0.02−0.36; *p* = 0.001), with a significant interaction between RV dysfunction status and treatment (*p* = 0.016). Results for sinus rhythm at 24 h and multiple recurrences were directionally consistent, though the latter did not reach statistical significance in this smaller subgroup. Full results are presented in eSupplementary Table S3.

A *post-hoc* subgroup analysis within the RV dysfunction group (*n* = 73) according to Covid-19 ARDS status showed directionally consistent treatment effects: arrhythmia recurrence occurred in 50.0% of propafenone-treated vs. 75.0% of amiodarone-treated patients in the Covid-19 ARDS subgroup (*n* = 18; OR 0.33, 95% CI 0.04−2.34; *p* = 0.287), and in 50.0% vs. 87.0% in the non-Covid subgroup (*n* = 55; OR 0.15, 95% CI 0.03−0.55; *p* = 0.008).

## Discussion

In this exploratory analysis focusing on right ventricular dysfunction in mechanically ventilated patients with septic shock and supraventricular arrhythmia, predominantly atrial fibrillation, treatment with the class Ic antiarrhythmic agent propafenone was associated with fewer arrhythmia recurrences compared with amiodarone. The study population was characterized by preserved to moderately reduced left ventricular systolic function and largely non-dilated left atria, a phenotype in which loss of sinus rhythm may be particularly poorly tolerated.

All patients were managed in a tightly controlled hemodynamic setting, including echocardiography-guided preload optimization in addition to standard invasive monitoring, with frequent use of adjunctive arginine vasopressin allowing reduction of norepinephrine doses. This standardized approach minimized confounding effects of volume status and catecholamine exposure on arrhythmia burden and rhythm control.

Although no statistically significant difference in short-term cardioversion rates was observed, propafenone was associated with a lower burden of recurrent arrhythmias, particularly repeated multiple sustained losses of sinus rhythm, in patients with right ventricular dysfunction. These findings suggest that propafenone may offer advantages in maintaining rhythm stability in this high-risk population, provided that left ventricular systolic function is not severely reduced and careful hemodynamic monitoring is ensured (19, 33, 34).

Supraventricular arrhythmias, particularly atrial fibrillation, are frequent complications in patients with septic shock requiring mechanical ventilation and are closely associated with right ventricular dysfunction. In our cohort, moderate-to-severe right ventricular dilatation, defined as an RV end-diastolic diameter–to–left ventricular end-diastolic diameter ratio ≥ 0.6, was observed in 171 mechanically ventilated patients with septic shock (81.8%).

Several mechanisms may contribute to this high prevalence. Volume expansion of the right ventricle, already exposed to non-physiological positive-pressure ventilation, may exacerbate right ventricular dilatation and increase right atrial pressure, thereby promoting atrial stretch and electrical instability. This pathophysiological milieu likely predisposes patients to the development and persistence of supraventricular arrhythmia during septic shock.

SVA, including atrial fibrillation, in the context of acute right ventricular dysfunction remain relatively underrepresented in both cardiology and intensive care literature. Our findings contribute to this gap by comparing rhythm-control strategies using propafenone and amiodarone in patients with septic shock and echocardiographically defined right ventricular dysfunction.

Previous studies have demonstrated the safety of the class Ic antiarrhythmic agent propafenone in patients with preserved or mildly to moderately reduced left ventricular systolic function, as well as its potential to achieve faster and more durable cardioversion compared with the widely used amiodarone (19). Our results extend these observations by suggesting that right ventricular dysfunction may represent an additional clinical context in which propafenone can be considered, provided that left ventricular systolic function is not severely impaired.

This observation challenges the broad and often imprecise application of the term “structural heart disease” in current guidelines, where its presence frequently limits pharmacological rhythm-control strategies to amiodarone. In critically ill patients, bedside echocardiography offers a practical means to refine phenotyping of ventricular function and may help distinguish patterns of cardiac involvement that are compatible with alternative antiarrhythmic strategies. Such an individualized, echocardiography-guided approach could potentially reduce reliance on amiodarone and limit exposure to its well-recognized multi-organ toxicity in the intensive care setting (35).

Amiodarone's established safety profile in severe LV systolic dysfunction and its role as the guideline-recommended antiarrhythmic agent in the setting of structural heart disease are well recognized. The key distinction in the present study is that severe LV dysfunction (LVEF <35%) was an explicit exclusion criterion: all patients had preserved to moderately reduced LV systolic function, which represents the specific clinical context in which class Ic agents such as propafenone may be considered. The negative chronotropic effect of propafenone - mediated through its beta-adrenergic blocking properties - may paradoxically be advantageous in patients with RV dysfunction and concomitant septic shock tachycardia driven hemodynamic compromise, where rate control itself may contribute to RV unloading. All patients in this study were managed in a tightly controlled hemodynamic setting with echocardiography-guided monitoring, which allowed early detection of any cardiac depressant effects and guided safe drug administration. Finally, it should be emphasized that these observations are specifically limited to mechanically ventilated patients with septic shock and do not extend to the post-cardiac surgery setting, where different substrate considerations apply including the effects of sternotomy, cardioplegia, and myocardial reperfusion with edema.

This study has several limitations that should be acknowledged. Echocardiographic assessment of right ventricular function could not be completed in 47 of the 209 patients originally enrolled in the trial, primarily due to limited acoustic windows in mechanically ventilated patients. A comparison of available baseline characteristics between patients with and without assessable RV function is provided in eSupplementary Table S4. Although three parameters differed statistically - randomization to amiodarone was more frequent, SOFA score was slightly higher, and serum magnesium was lower in the non-assessable group - age, sex, arrhythmia type, illness severity (APACHE II), vasopressor requirements, and all other laboratory parameters were comparable between groups, suggesting that missing echocardiographic data were largely random with respect to clinical characteristics. Nevertheless, the possibility of selection bias cannot be fully excluded (36). Moreover, inclusion of the patients with moderate RV dilatation and venous congestion, which were frequently observed among the mechanically ventilated patients with septic shock (32), could have been related to an observed non-significant relationship to supraventricular arrhythmias. Limiting the definition of RV dysfunction to severely dilated RV, signs of ACP or RV-pulmonary artery pressure uncoupling could have proven the relationship similarly to other published data (6, 7). A sensitivity analysis using a strict definition limited to the four primary criteria - without IVC-based adjudication - yielded directionally consistent and numerically stronger results for the primary treatment comparison, supporting the robustness of the main findings (eSupplementary Table S3).

A proportion of patients classified as having right ventricular dysfunction (18 of 73, 24.7%) had SARS-CoV-2-associated acute respiratory distress syndrome, a rate comparable to that observed in the control group without RV dysfunction (18 of 89, 20.2%; *p* = 0.5). In patients with Covid-19-associated ARDS, right ventricular dysfunction is primarily driven by pulmonary parenchymal and vascular disease rather than stress cardiomyopathy *per se*, and we are unable to fully disentangle these mechanisms in the current dataset. The treatment effect of propafenone relative to amiodarone was directionally consistent across both Covid-19 ARDS and non-Covid subgroups. While supraventricular arrhythmias have been reported in 17%–25% of patients with severe, mechanically ventilated Covid-19 and have been associated with adverse outcomes (37, 38), such an association was not observed in our cohort, as 12-month survival did not differ between patients with right ventricular dysfunction and controls.

Furthermore, multiple outcomes were assessed without correction for multiplicity, which increases the risk of type I error; all results should therefore be interpreted in the context of the exploratory design. *post-hoc* power calculations indicated adequate power (84.8%) for the primary outcome of arrhythmia recurrence based on the observed effect size (propafenone 50.0% vs. amiodarone 83.9%; Cohen's h = 0.745), but lower power (59.3%) for the secondary outcome of multiple recurrences, which should be interpreted accordingly.

In conclusion, this exploratory analysis is hypothesis-generating and suggests that antiarrhythmic agents with beta-receptor–blocking properties other than amiodarone may provide more effective rhythm control in critically ill patients with right ventricular dysfunction. Among these agents, propafenone was associated with fewer arrhythmia recurrences compared with amiodarone and did not demonstrate clinically relevant cardiac toxicity when therapy was guided by echocardiography, even in the presence of moderate structural heart disease (19) (39–41).

These findings support further prospective studies evaluating the efficacy and safety of different rhythm-control strategies in critically ill patients with supraventricular arrhythmias and right ventricular dysfunction.

## Data Availability

The datasets presented in this study can be found in online repositories and upon reasonable request to the corresponding author. The names of the repository/repositories and accession number(s) can be found in the article/Supplementary Material.
