# Supplementary material for: Propafenone is associated with fewer recurrences of supraventricular arrhythmias in mechanically ventilated patients with septic shock and right ventricular dysfunction
Source: Front Cardiovasc Med. 2026 May 7;13:1807380. doi: 10.3389/fcvm.2026.1807380 (PMC13191741; doi:10.3389/fcvm.2026.1807380)
Supplement: Supplementary file 1 [file Datasheet1.docx]

**Electronic Supplementary Material (ESM) for:**

**Propafenone is associated with fewer recurrences of supraventricular arrhythmias in mechanically ventilated patients with septic shock and right ventricular dysfunction**

**eTable 1.** **Additional baseline characteristics of patients stratified by right ventricular dysfunction.** Data are presented as median (interquartile range) or number (percentage), as appropriate. Comparisons between groups were performed using the Wilcoxon rank-sum test for continuous variables and Pearson’s chi-squared test or Fisher’s exact test for categorical variables. Abbreviations: HR, heart rate; BPs, systolic blood pressure; BPd, diastolic blood pressure; BPmean, mean arterial pressure; CVP, central venous pressure; CRRT, continuous renal replacement therapy; IPPV, invasive positive-pressure ventilation; ASV, adaptive support ventilation; PSV, pressure support ventilation; BIPAP, bilevel positive airway pressure; SIMV, synchronized intermittent mandatory ventilation; MMV, mandatory minute ventilation.

| **Parameter** | **All patients**  **n=162** | **RV dysfunction**  **n=73** | **Control group**  **n=89** |
| --- | --- | --- | --- |
| HR (beats/min) | 130 (111, 145) | 130 (119, 150) | 130 (110, 145) |
| BPs (mmHg) | 110 (105, 125) | 115 (100, 125) | 110 (105, 125) |
| BPd (mmHg) | 56 (50, 60) | 56 (50, 63) | 55 (50, 60) |
| BPmean (mmHg) | 75 (70, 80) | 75 (69, 81) | 75 (70, 80) |
| Urea (mmol/L) | 18 (11, 24) | 16 (11, 23) | 19 (11, 25) |
| Creatinine ($\mu$mol/L) | 154 (94, 229) | 130 (85, 222) | 157 (107, 230) |
| Total bilirubin (µmol/L) | 16 (11, 29) | 16 (11, 28) | 17 (11, 30) |
| Conjugated bilirubin (µmol/L) | 9 (6, 19) | 9 (6, 15) | 9 (6, 20) |
| ALT (µkat/L) | 0.7 (0.4, 1.8) | 0.7 (0.4, 1.5) | 0.7 (0.5, 2.4) |
| AST (µkat/L) | 1.0 (0.6, 2.2) | 0.8 (0.5, 2.1) | 1.1 (0.7, 2.4) |
| GGT (µkat/L) | 1.69 (0.80, 3.40) | 1.49 (0.74, 2.61) | 1.95 (0.92, 3.69) |
| ALP (µkat/L) | 1.72 (1.19, 2.59) | 1.58 (1.16, 2.22) | 1.84 (1.37, 2.67) |
| Hypothyroidism | 16 (9.9%) | 7 (9.6%) | 9 (10.1%) |
| Euthyroid on medication | 16 (9.9%) | 7 (9.6%) | 9 (10.1%) |
| Hyperthyroidism | 1 (0.6%) | 0 (0.0%) | 1 (1.1%) |
| CRRT | 27 (16.7%) | 9 (12.3%) | 18 (20.2%) |
| ***Invasive ventilation mode*** | | | |
| ASV | 9 (5.6%) | 5 (6.8%) | 4 (4.5%) |
| PSV | 55 (34.0%) | 19 (26.0%) | 36 (40.4%) |
| BIPAP | 45 (27.8%) | 24 (32.9%) | 21 (23.6%) |
| SIMV | 44 (27.2%) | 20 (27.4%) | 24 (27.0%) |
| MMV | 3 (1.9%) | 1 (1.4%) | 2 (2.2%) |

**eTable 2.** **Prior medication history, concomitant potentially proarrhythmogenic drugs, and additional antiarrhythmic therapies administered during the ICU stay.**

Data are presented as numbers (percentages). Comparisons between groups were performed using Pearson’s chi-squared test or Fisher’s exact test, as appropriate. Clarithromycin was the only medication that differed significantly between groups (p = 0.046).

* The reported rates of beta-blocker use represent the proportion of patients receiving metoprolol at the end of the study medication period and continuing beta-blockade after discontinuation of the study infusion.

| **Parameter** | **All patients**  **(n=162)** | **RV dysfunction**  **n=73** | **Control group n=89** |
| --- | --- | --- | --- |
| ***A history of*** | | | |
| Beta-blocker | 40 (24.7%) | 19 (26.0%) | 21 (23.6%) |
| Amiodarone | 12 (7.4%) | 6 (8.2%) | 6 (6.7%) |
| Calcium channel blocker | 15 (9.3%) | 9 (12.3%) | 6 (6.7%) |
| Digoxin | 2 (1.2%) | 2 (2.7%) | 0 (0.0%) |
| Propafenone | 5 (3.1%) | 2 (2.7%) | 3 (3.4%) |
| Chemotherapy within the last 3 months | 23 (14.2%) | 11 (15.1%) | 12 (13.5%) |
| Anthracycline chemotherapy | 2 (1.2%) | 1 (1.4%) | 1 (1.1%) |
| ***ICU medications*** | | | |
| Haloperidol | 10 (6.2%) | 6 (8.2%) | 4 (4.5%) |
| **Clarithromycin** | **7 (4.3%)** | **6 (8.2%)** | **1 (1.1%)** |
| Fluoroquinolone | 30 (18.5%) | 15 (20.5%) | 15 (16.9%) |
| Amphotericin B | 22 (13.6%) | 9 (12.3%) | 13 (14.6%) |
| Azole antifungal | 54 (33.3%) | 27 (37.0%) | 27 (30.3%) |
| Anticholinergics | 1 (0.6%) | 0 (0.0%) | 1 (1.1%) |
| Aminophylline | 0 (0.0%) | 0 (0.0%) | 0 (0.0%) |
| Terbutaline | 1 (0.6%) | 0 (0.0%) | 1 (1.1%) |
| ***Additional antiarrhythmic therapy*** | | | |
| Beta-blocker* | 39 (24.1%) | 21 (28.8%) | 18 (20.2%) |
| Magnesium | 47 (29.0%) | 23 (31.5%) | 24 (27.0%) |
| Digoxin | 8 (4.9%) | 3 (4.1%) | 5 (5.6%) |
| Adenosine | 0 (0.0%) | 0 (0.0%) | 0 (0.0%) |
| Verapamil | 1 (0.6%) | 0 (0.0%) | 1 (1.1%) |
| Lidocaine | 1 (0.6%) | 0 (0.0%) | 1 (1.1%) |

**eTable 3.** **Sensitivity analysis using a strict definition of right ventricular (RV) dysfunction.** Under this definition, RV dysfunction was classified based on the four primary echocardiographic criteria only (PASP >40 mmHg with TAPSE <15 mm; PASP >40 mmHg with paradoxical septal motion; TAPSE <15 mm with paradoxical septal motion; RV/LV EDD ratio ≥1.0), without the IVC-based adjudication step applied to patients with moderate RV dilatation (RV/LV EDD ratio 0.6-1.0). Under this stricter definition, RV function could be classified in 110 patients (56.1% of those who received treatment): 60 patients met criteria for RV dysfunction (36 propafenone, 24 amiodarone) and 50 served as controls (26 propafenone, 24 amiodarone). Data are presented as n (%). Comparisons were performed using Pearson’s chi-squared test or Fisher’s exact test. Odds ratios (ORs) with 95% confidence intervals (CIs) were calculated using logistic regression.

| **Outcome** | **RV dysfunction** | | **OR (95% CI)** | **p-value** |
| --- | --- | --- | --- | --- |
|  | **Propafenone n=36** | **Amiodarone n=24** |  |  |
| Sinus rhythm at 24 hours | 26 (72.2%) | 12 (50.0%) | 0.38 (0.13-1.12) | 0.080 |
| Arrhythmia recurrence | 15 (41.7%) | 21 (87.5%) | 0.10 (0.02-0.36) | <0.001 |
| Multiple (>3) recurrences* | 4 (26.7%) | 9 (42.9%) | 0.48 (0.11-1.96) | 0.300 |
| Interaction: RV dysfunction x treatment (recurrence)† |  |  | 9.26 (1.61-63.3) | 0.016 |

* Among patients with at least one recurrence (propafenone n=15, amiodarone n=21).

† Interaction term from a logistic regression model including RV dysfunction status, treatment group, and their product term (RV dysfunction x Amiodarone as reference category). OR >1 indicates that the treatment effect of propafenone relative to amiodarone on arrhythmia recurrence is greater in patients with RV dysfunction than in those without.

Abbreviations: CI, confidence interval; IVC, inferior vena cava; OR, odds ratio; PASP, pulmonary artery systolic pressure; RV, right ventricular; LV, left ventricular; EDD, end-diastolic diameter; TAPSE, tricuspid annular plane systolic excursion.

**eTable 4.** **Comparison of baseline characteristics between patients with assessable right ventricular (RV) function (n=162) and those in whom RV assessment was not possible (n=34), among the 196 patients who received antiarrhythmic treatment.** Data are presented as mean (SD); median (IQR) for continuous variables and n (%) for categorical variables. Comparisons were performed using the Wilcoxon rank-sum test for continuous variables and Pearson's chi-squared test or Fisher's exact test for categorical variables, as appropriate. Three parameters differed significantly between groups: randomization to amiodarone was more frequent among patients with non-assessable RV function (67.6% vs. 46.9%, p=0.028), SOFA score was slightly higher (median 11.5 vs. 10.0, p=0.025), and serum magnesium was lower (median 1.07 vs. 1.30 mmol/l, p=0.004) in the non-assessable group. All other baseline characteristics, including age, sex, arrhythmia type, illness severity (APACHE II), vasopressor requirements, and laboratory parameters, did not differ significantly between groups. Abbreviations: SVT, supraventricular tachycardia; AF, atrial fibrillation; CVP, central venous pressure; SOFA, Sequential Organ Failure Assessment; APACHE II, Acute Physiology and Chronic Health Evaluation II.

| **Parameter** | **N** | **All patients N = 196** | **Assessable N = 162** | **Not assessable N = 34** | **p-value** |
| --- | --- | --- | --- | --- | --- |
| Study group | 196 |  |  |  | 0.028 |
| Amiodarone |  | 99.0 (50.5%) | 76.0 (46.9%) | 23.0 (67.6%) |  |
| Propafenone |  | 97.0 (49.5%) | 86.0 (53.1%) | 11.0 (32.4%) |  |
| Age (years) | 196 | 68 (12); 70 (62, 76) | 68 (12); 70 (63, 76) | 67 (13); 69 (59, 78) | 0.8 |
| Sex | 196 |  |  |  | 0.8 |
| Female |  | 77.0 (39.3%) | 63.0 (38.9%) | 14.0 (41.2%) |  |
| Male |  | 119.0 (60.7%) | 99.0 (61.1%) | 20.0 (58.8%) |  |
| BMI | 196 | 31 (8); 29 (26, 33) | 31 (8); 29 (26, 33) | 30 (7); 28 (24, 32) | 0.3 |
| SOFA | 196 | 10.21 (2.97); 10.00 (8.0, 12.0) | 9.99 (2.94); 10.00 (8.0, 12.0) | 11.29 (2.92); 11.50 (9.0, 13.0) | 0.025 |
| APACHE II | 196 | 25 (7); 25 (20, 30) | 25 (7); 25 (20, 30) | 26 (7); 25 (21, 30) | 0.6 |
| History of paroxysmal SVA | 196 |  |  |  | 0.11 |
| No |  | 160.0 (81.6%) | 129.0 (79.6%) | 31.0 (91.2%) |  |
| Yes |  | 36.0 (18.4%) | 33.0 (20.4%) | 3.0 (8.8%) |  |
| El. CV prior to inclusion | 196 |  |  |  | 0.4 |
| No |  | 171.0 (87.2%) | 143.0 (88.3%) | 28.0 (82.4%) |  |
| Yes |  | 25.0 (12.8%) | 19.0 (11.7%) | 6.0 (17.6%) |  |
| Arrhythmia type | 196 |  |  |  | 0.7 |
| AF |  | 149.0 (76.0%) | 124.0 (76.5%) | 25.0 (73.5%) |  |
| Flutter |  | 39.0 (19.9%) | 32.0 (19.8%) | 7.0 (20.6%) |  |
| SVT |  | 8.0 (4.1%) | 6.0 (3.7%) | 2.0 (5.9%) |  |
| Norepinephrine (µg/kg min) | 196 | 0.34 (0.27); 0.30 (0.15, 0.44) | 0.35 (0.27); 0.30 (0.16, 0.45) | 0.29 (0.24); 0.22 (0.10, 0.41) | 0.12 |
| Dobutamine (µg/kg min) | 10 | 2.95 (1.34); 3.00 (2.50, 3.00) | 2.95 (1.34); 3.00 (2.50, 3.00) | NA |  |
| Unknown |  | 186 | 152 | 34 |  |
| Arginine vasopressin (IU/h) | 52 | 2.34 (1.02); 2.00 (2.00, 3.00) | 2.38 (1.04); 2.00 (2.00, 3.00) | 1.90 (0.50); 2.00 (1.60, 2.20) | 0.6 |
| Unknown |  | 144 | 114 | 30 |  |
| Potassium (mmol/l) | 196 | 4.34 (0.52); 4.30 (4.00, 4.60) | 4.35 (0.50); 4.30 (4.10, 4.60) | 4.30 (0.59); 4.25 (4.00, 4.60) | 0.5 |
| Calcium (mmol/l) | 193 | 1.92 (0.24); 1.92 (1.80, 2.05) | 1.91 (0.22); 1.91 (1.79, 2.03) | 1.94 (0.29); 1.95 (1.80, 2.09) | 0.4 |
| Unknown |  | 3 | 3 | 0 |  |
| Ionized calcium (mmol/l) | 196 | 1.04 (0.17); 1.05 (0.95, 1.12) | 1.03 (0.18); 1.05 (0.94, 1.11) | 1.05 (0.15); 1.09 (0.98, 1.14) | 0.14 |
| Magnesium (mmol/l) | 196 | 1.31 (0.40); 1.25 (1.02, 1.56) | 1.34 (0.39); 1.30 (1.10, 1.57) | 1.17 (0.41); 1.07 (0.86, 1.24) | 0.004 |
| Hb (g/l) | 196 | 105 (21); 101 (89, 119) | 104 (20); 101 (89, 117) | 110 (28); 106 (92, 130) | 0.3 |
| WBC (103/µl) | 196 | 21 (37); 17 (13, 25) | 22 (40); 18 (13, 25) | 17 (12); 14 (10, 23) | 0.079 |
| pH art | 196 | 7.35 (0.08); 7.36 (7.30, 7.41) | 7.35 (0.08); 7.36 (7.30, 7.41) | 7.37 (0.08); 7.39 (7.31, 7.43) | 0.11 |
| pO_2_ art (kPa) | 196 | 18 (24); 11 (10, 14) | 18 (25); 11 (10, 14) | 17 (18); 11 (10, 14) | 0.7 |
| pCO_2_ art (kPa) | 196 | 9 (11); 6 (6, 8) | 9 (10); 7 (6, 8) | 9 (11); 6 (5, 8) | 0.15 |
| BE art (mmol/l) | 196 | 1 (6); 1 (-3, 5) | 1 (6); 2 (-2, 5) | 1 (7); 1 (-5, 5) | 0.9 |
| HCO_3_ art (mmol/l) | 196 | 27 (7); 27 (23, 31) | 27 (7); 27 (23, 31) | 26 (7); 25 (22, 30) | 0.4 |
| Lactate art (mmol/l) | 196 | 3.71 (3.30); 2.60 (2.20, 3.60) | 3.87 (3.58); 2.55 (2.20, 3.80) | 2.95 (0.96); 2.65 (2.30, 3.40) | >0.9 |
| CRP (mg/l) | 196 | 215 (130); 193 (105, 315) | 213 (128); 187 (102, 316) | 224 (142); 215 (147, 311) | 0.8 |
| PCT (nmol/ml) | 195 | 24 (58); 4 (1, 18) | 23 (59); 3 (1, 17) | 29 (58); 6 (1, 26) | 0.2 |
| Unknown |  | 1 | 0 | 1 |  |
